# Field efficacy and safety evaluation of a novel chewable tablet (Credelio Quattro™) containing lotilaner, moxidectin, praziquantel, and pyrantel against gastrointestinal nematode infections in dogs in the USA

**DOI:** 10.1186/s13071-025-06932-7

**Published:** 2025-07-25

**Authors:** Samuel Charles, Scott Wiseman, Molly D. Savadelis, Eric Nichols, Lisa Young

**Affiliations:** 1 Elanco Animal Health, 2500 Innovation Way, Greenfield, IN 46140 USA; 2 Elanco Animal Health, Bartley Way, Hook, Hants RG27 9XA UK

**Keywords:** Credelio Quattro, Hookworms, Roundworms, *Ancylostoma caninum*, *Toxocara canis*

## Abstract

**Background:**

Gastrointestinal nematodes such as hookworms (*Ancylostoma caninum*) and roundworms (*Toxocara canis*) commonly infect dogs and are zoonotic parasites capable of producing clinical disease in humans. This field study was conducted to confirm the clinical effectiveness and field safety of a novel, chewable tablet (Credelio Quattro^™^) containing lotilaner, moxidectin, praziquantel, and pyrantel (as pamoate salt) as compared with a control product containing sarolaner, moxidectin, and pyrantel (as pamoate salt) for the treatment of naturally occurring gastrointestinal nematode infections in dogs.

**Methods:**

In this multicenter field study, 158 dogs were enrolled with an evaluable safety population of 155 dogs and an evaluable effectiveness population of 109 dogs. Dogs were randomized in a 2:1 ratio to receive either the investigational veterinary product (IVP) Credelio Quattro containing lotilaner, moxidectin, praziquantel, and pyrantel or the control product (CP) containing sarolaner, moxidectin, and pyrantel. On Day 0, after confirmation of infection, enrolled dogs were randomized, dispensed, and administered IVP or CP. Enrolled dogs returned to the study site on Day 10 (± 2 d) to quantify the concentration of nematode eggs post-treatment.

**Results:**

Post-treatment fecal egg counts performed on Day 10 demonstrated a 99.9% reduction in *A. caninum* eggs per gram in both IVP-treated dogs and CP-treated dogs. In addition, a 98.7% and 96.6% reduction in *T. canis* fecal egg counts were observed in IVP- and CP-treated dogs, respectively. Adverse events were reported in both treatment groups. The most common adverse events reported in the IVP group included gastrointestinal signs, such as diarrhea and emesis, as well as lethargy.

**Conclusions:**

This field study demonstrated that Credelio Quattro, a novel oral combination chewable tablet administered at the labeled dose ranges of 20–40 mg/kg lotilaner, 0.02–0.04 mg/kg moxidectin, 5–10 mg/kg praziquantel, and 5–10 mg/kg pyrantel (as pamoate salt), is safe and effective for the treatment and control of naturally occurring *A. caninum* and *T. canis* infections in dogs.

**Graphical Abstract:**

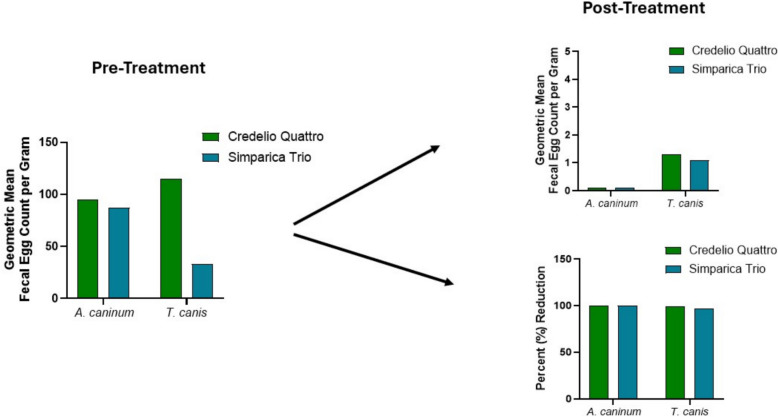

## Background

Gastrointestinal parasites in dogs such as *Toxocara canis* (roundworms) and *Ancylostoma caninum* (hookworms) in the USA are some of the most important and commonly diagnosed infections. In a study evaluating monthly prevalence data captured by the Companion Animal Parasite Council (CAPC) in the USA between 2012 and 2018, prevalence rates of *A. caninum* were reported to have increased yearly, with an overall 47% increase during this time [1]. Yearly prevalence rates for *T. canis* remained relatively consistent while *Trichuris vulpis*, whipworm, was found to have decreasing prevalence from 0.8% to 0.67% between 2012 and 2018 [1]. In Colorado, a study found the prevalence of canine hookworm increased by 137.3%, and roundworm increased by 35.6%, from 2013 to 2017, highlighting the important role dog importation plays in parasite and disease transmission, with many shelters and rescue organizations relocating dogs from the southeastern USA [2].

Dogs, the definitive host for *T. canis*, become infected via transplacental transmission (in utero) or by ingesting larvated eggs or infected paratenic hosts such as rodents, rabbits, chickens, and other birds [3–5]. Eggs shed in the environment require 2–4 weeks to develop to the infective third-stage larvae (L3) [6]. Roundworm eggs are extremely resistant to environmental changes, capable of surviving for many years owing to their thick outer shell, therefore timely removal of infected fecal material from the environment is key to reducing transmission [7, 8]. After ingestion of larvated eggs, roundworm penetrate the intestinal mucosa, migrate through the liver and lungs, are coughed up and re-ingested, finally residing in the small intestine and developing into sexually mature adult worms [6]. In adult dogs, many ingested roundworm larvae arrest in somatic tissues during migration and are then activated during pregnancy, infecting puppies in utero transplacentally and, to a lesser degree, through transmammary transmission while nursing [9].

*Ancylostoma caninum* infects dogs through ingestion of larvae from the environment, direct larval skin penetration, or ingestion of infected paratenic hosts such as rodents [10–12]. Hookworm eggs hatch and develop to L3 after approximately 2–9 days in the environment depending on the temperature and humidity, with hookworm being more susceptible to desiccation than roundworm. After ingestion, some L3 migrate through the lungs and arrest in somatic tissue, while other L3 remain in the alimentary tract and develop into sexually mature adults in the small intestine [13]. If infected by direct skin penetration, L3 migrate via the veins and lymphatics to the lungs, enter the trachea where the parasite is swallowed, and then develop in the small intestine to sexually mature adults [12]. In addition, transmammary infection can occur as arrested larvae in the tissues are reactivated during pregnancy and migrate to the mammary glands, thereby infecting puppies during nursing [14]. Arrested larvae in the somatic tissues can reactivate in dogs outside of pregnancy and can continuously migrate to the small intestine and develop to the adult stage leading to repeated repopulation of the intestine and prolonged egg shedding despite treatment, termed “larval leak” [15].

While many roundworm and hookworm infections in dogs may be asymptomatic, the most common clinical signs include diarrhea and vomiting, abdominal distention, and lack of growth in young dogs in roundworm infections, as well as anemia, malnutrition, and even death in severe hookworm infections [10, 16]. In addition, roundworms and hookworms are important zoonotic parasites. Infection in humans with *T. canis* can cause visceral larva migrans (VLM), ocular larva migrans, or neurotoxocariasis with resulting brain tissue damage, and infection with *Ancylostoma* spp. can induce cutaneous larva migrans (CLM) [17–19].

In order to properly diagnose and treat these important gastrointestinal parasites, CAPC currently recommends dogs receive fecal examinations at least four times in their first year of life and then at least twice annually thereafter, by fecal flotation with centrifugation. Puppies should be treated with anthelmintics at 2-week intervals, starting at 2 weeks of age until 8 weeks of age, and then administration of a year-round broad-spectrum anthelmintic thereafter to provide continuous protection [20]. In addition, prompt removal of feces from the environment is crucial to help prevent environmental contamination and future transmission. Drugs currently labeled for the treatment of *T. canis* and/or *A. caninum* by the US Food and Drug Administration (FDA) include fenbendazole, febantel, pyrantel, milbemycin oxime, and moxidectin, available in many different administration routes such as oral, topical, and injectable, and developed as both monotherapy and combination therapy formulations. Incorporation of combination therapy products in veterinary clinics has been demonstrated to increase the average number of monthly doses dispensed to clients as compared with monotherapy products, thereby increasing parasite coverage [21]. In addition, the ease of use of combination therapy products may increase owner compliance with timely and adequate administration.

The occurrence of drug resistance in *A. caninum* has been documented recently in racing greyhound and pet dog populations across the USA [22–24]. Fecal samples obtained from suspected *A. caninum-*resistant cases were evaluated for benzimidazole and macrocyclic lactone resistance in vitro using the egg hatch (EHA) and larval development (LDA) assays, and pyrantel resistance in vivo, demonstrating in vitro resistance ratios ranging from 6.0 to > 100 and 5.5 to 69.8 for the EHA and LDA, respectively. No reduction in fecal egg counts were observed post-treatment with pyrantel, providing additional confirmation these hookworm isolates were multidrug resistant [25]. The mechanism of action and corresponding mutations associated with benzimidazole resistance in *A. caninum* has been confirmed, with a commercial diagnostic test available to detect molecular markers in fecal samples (KeyScreen^®^ GI Parasite PCR, Antech Diagnostics, Inc.). This information is not yet understood for macrocyclic lactones and pyrantel, requiring in vitro EHA or LDA to identify macrocyclic lactone resistance and in vivo fecal egg count reduction testing to identify pyrantel resistance [23].

This real-world field study provides practical information on product performance for veterinarians and pet owners, especially given rising *A. caninum* resistance. The objective of the field study described below was to evaluate the efficacy and safety of a novel, oral chewable tablet containing lotilaner, moxidectin, praziquantel, and pyrantel (Credelio Quattro^™^, Elanco Animal Health, Greenfield, IN, USA), against naturally occurring roundworm and hookworm infections in dogs in the USA.

## Methods

This multicenter field study was conducted according to VICH GL9 Good Clinical Practice, VICH GL19 Effectiveness of Anthelmintics: Specific Recommendations for Canines, and VICH GL7 Effectiveness of Anthelmintics: General Recommendations [26]. The protocol was reviewed and approved by the Elanco Animal Care and Use Committee (IACUC no. 2008) prior to initiation of the study and owner consent was obtained prior to eligibility screening and enrollment.

At the time of eligibility screening, a brief medical history and fecal sample (≥ 5 g) for a quantitative fecal egg count (FEC) of gastrointestinal nematode eggs were obtained. For dogs ≥ 6 months of age, a blood sample was also collected for detection of the presence of heartworm antigen and circulating microfilaria by modified Knott’s test.

To be enrolled in the study, dogs were required to be at least 8 weeks old and weigh at least 3.3 lbs. at the time of dosing (Day 0). Any domestic breed, male or female, spayed, neutered, or intact dogs were permitted, provided they were not pregnant, lactating, or intended for breeding during the study. A positive test result for nematode infection was required, with a minimum of 10 eggs/g (EPG) of feces, specifically for *A. caninum*, *T. canis*, *Uncinaria stenocephala*, and/or *Toxascaris leonina*. Dogs had not received a long-acting topical or injectable parasiticide within 6 months of Day 0. The examining veterinarian had to deem the dog to be in good general health. If the dog was ≥ 6 months old, a negative test result for heartworm antigen and microfilariae was required. All dogs within a multi-animal household were screened for eligibility requirements but only one dog in the household was enrolled in the study.

### Animals

Domestic dogs in good health after a physical examination at enrollment and meeting all inclusion criteria were enrolled in this field study. Food and water were provided according to normal practice and dogs remained with their owners throughout the study unless they required care by a qualified veterinarian as authorized by the owner. Administration of routine vaccinations were permitted as necessary during the study.

### Randomization and treatment

Dogs were randomized in a 2:1 ratio, in blocks of six dogs, to receive either the investigational veterinary product (IVP) Credelio Quattro containing 20–40 mg/kg lotilaner, 0.02–0.04 mg/kg moxidectin, 5–10 mg/kg praziquantel, and 5–10 mg/kg pyrantel, or a control product (CP) (Simparica Trio^®^, Zoetis Parsippany, NJ, USA) containing 1.2–2.4 mg/kg sarolaner, 0.024–0.048 mg/kg moxidectin, and 5–10 mg/kg pyrantel. All study site personnel were masked to treatment group designations except the treatment dispenser and owner.

On Day 0, according to the randomization, enrolled dogs were dispensed and administered IVP or CP on the basis of Day 0 body weights. Administration of IVP was performed within approximately 30 min after the dog’s last meal. CP was administered either with or without food according to the product label.

### Fecal egg counts

Fecal samples (≥ 5 g) were obtained prior to enrollment to confirm eligibility and on Day 10 (± 2 d) and were examined by quantitative centrifugation (Modified Wisconsin) for the presence of gastrointestinal nematode eggs. Samples were collected directly from the dog or as a freshly voided sample, refrigerated, and shipped to a central laboratory (TRS Labs, Inc, Athens, GA, USA; IDEXX BioAnalytics, West Sacramento, CA, USA) for processing. Briefly, feces were mixed with flotation solution, strained into a 15 mL centrifuge tube filling the tube within approximately 2–3 mm of the top and centrifuged at 500 *g* for 5 min. After centrifugation, additional flotation solution was added to samples until a slight meniscus formed, a coverslip was placed on the centrifuge tube and allowed to sit for 10 min before examination. An additional wash step with water may have been performed prior to centrifugation with flotation solution.

### Safety assessments

All enrolled dogs that received a dose of either IVP or CP were evaluated for safety. Any observation in an animal or human, whether it was considered to be product related or not, that was unfavorable or unintended that occurred after dose administration of either IVP or CP was defined as an adverse event and documented. All concurrent medications administered after dosing on Day 0 were documented. Medications or therapies that could directly affect the evaluation of the IVP or CP were not allowed during the study, such as the use of drugs with endoparasitic efficacy. Drugs with antiprotozoal activity only were allowed if necessary.

### Statistical analysis

All statistical analysis was performed using SAS version 9.4 (SAS Institute, Cary, NC, USA) with an alpha level of 0.05 using two-tailed testing. The fecal egg count reduction from pretreatment to 10 d post-treatment was calculated to assess the effect of treatment with IVP or CP. Analysis was conducted for each parasite species with ≥ 10 evaluable cases. Fecal egg counts were natural log transformed and analyzed using a linear mixed model with dog identification number as a random effect and time-point as the fixed effect. Mean percent fecal egg count reduction was calculated using the following formula, [(pretreatment GM mean − post-treatment GM mean)/pretreatment GM] × 100. Geometric means (GM) were obtained by back-transforming the model least squares (LS) means.

## Results

In this multicenter field study, 158 dogs (IVP = 105, CP = 53) were enrolled with an evaluable safety population of 155 dogs (IVP = 103, CP = 52) and an evaluable effectiveness population of 109 dogs (IVP = 72, CP = 37). Three dogs were removed after randomization prior to treatment administration due to voluntarily owner removal. In total, 27 sites throughout the USA participated in this field study, with 17 sites successfully enrolling dogs. Population demographic data for all dogs in the safety population is summarized in Table 1, with similar demographics between experimental groups reported.

**Table 1:** Safety population demographics

Demographic	IVP (*n* = 103)	CP (*n* = 52)
Age, mean (months)	12.0	10.2
Age, range (months)	1–119	1–60
Body weight, mean (kg)	12.42	13.20
Body weight, range (kg)	1.8–41.9	2.3–33.6
Female (*n*)	53	27
Female, intact (*n*)	48	24
Male (*n*)	50	25
Male, intact (*n*)	47	24
Purebred (*n*)	37 (35.9%)	20 (38.5%)
Mixed breed (*n*)	42 (40.8%)	18 (34.6%)
Unknown (*n*)	24 (23.3%)	14 (26.9%)

### Safety

Concomitant medications were administered in 45% of IVP-treated dogs and 37% of CP-treated dogs. Vaccines were the most commonly reported concomitant medication administered, followed by antimicrobials and various otic topicals. Common pre-existing conditions included ectoparasitic infestations, protozoal infections, dermatologic disorders, and congenital abnormalities such as hernias, prolapsed nictitating membrane, cryptorchidism, and entropion.

One serious adverse event was reported that was not considered related to treatment, in which a dog was hit by a car and died 8 d post-dose in the IVP-treated group. The animal rate of post-treatment adverse events was 19.4% in the IVP-treated group and 5.8% in the CP-treated group. Diarrhea was the most commonly reported adverse event for both treatment groups (8.7% IVP and 3.8% CP). Additional adverse events reported in dogs in the IVP group were flatulence (3.9%), emesis (3.9%), and lethargy (2.9%).

### Fecal egg count reduction

Dogs that completed the study and were observed not to have major protocol deviations were evaluated in the effectiveness population (IVP = 72, CP = 37). Overall, 80 dogs were diagnosed positive for infection with *A. caninum* and 45 dogs with *T. canis*; 10 dogs were co-infected with *A. caninum* and *T. canis*. Of the 109 dogs within the effectiveness evaluable population, 22 dogs were also infected with *T. vulpis*, and one dog was infected with *U. stenocephala*. Post-treatment mean fecal egg count reduction was statistically significant (*P* < 0.0001) compared with pretreatment mean counts for IVP-treated dogs against both *A. caninum* and *T. canis* infections with 99.9 and 98.7% reductions, respectively (Table 2). A 99.9% and 96.6% reduction in mean fecal egg count were observed against *A. caninum* and *T. canis*, respectively, in the CP-treated group (*P* ≤ 0.0001).

**Table 2:** Efficacy of a single oral dose of Credelio Quattro against *Ancylostoma caninum* and *Toxocara canis* infections in dogs 10 d post-treatment

			Geometric mean fecal egg count (EPG)		Efficacy	
Treatment group	Parasite	*n*	Pretreatment mean (range)	Post-treatment mean (range)	% Reduction	*P*-value
IVP	*A. caninum*	52	99.9 (10–5019)	0.1 (0–22)	99.9	*P* < 0.0001 (t_53_ = 19.89)
CP	*A. caninum*	27	86.3 (10–1393)	0.1 (0–15)	99.9	*P* < 0.0001 (t_27_ = 17.01)
IVP	*T. canis*	33	109.4 (10–1901)	1.4 (0–136)	98.7	*P* < 0.0001 (t_33_ = 12.16)
CP	*T. canis*	11	33.1 (10–100)	1.1 (0–198)	96.6	*P* = 0.0001 (t_10_ = 6.02)

## Discussion

Roundworms and hookworms are the most commonly diagnosed gastrointestinal nematode parasite infections in dogs and are of great clinical importance owing to the zoonotic potential to humans. This multisite field study in the USA demonstrated efficacy against these important species with a 99.9% reduction in fecal egg count against *A. caninum* and a 98.7% reduction in fecal egg count against *T. canis* after a single dose of Credelio Quattro.

These results are consistent with and support findings from laboratory efficacy studies against both *T. canis* and *A. caninum* for Credelio Quattro. In experimentally infected animals Credelio Quattro demonstrated ≥ 97% efficacy against both immature adult and adult stages of *T. canis* under laboratory conditions [27]. In other experimentally infected animal studies, Credelio Quattro demonstrated ≥ 99.0% efficacy against *A. caninum* fourth-stage larvae, immature adults (L5), and adult stages, exhibiting robust protection against this species [28]. In addition, while a sufficient number of *T. leonina* and *U. stenocephala* infections were not diagnosed for statistical analysis in this field study, previous laboratory studies with Credelio Quattro also demonstrated 100% efficacy against adult *T. leonina* and ≥ 99.6% efficacy against adult *U. stenocephala* [27].

Within this field study, *T. canis* fecal egg count percent reductions were higher in dogs treated with Credelio Quattro (98.7%) compared with CP-treated dogs (96.6%), although this difference was not statistically compared. At 10 d post-treatment, only one dog per treatment group demonstrated a fecal egg count reduction of < 95% for *A. caninum* (≤ 64%). Both dogs were at least 1 year old. While larval leak or resistance to macrocyclic lactones and/or tetrahydropyrimidines might explain the incomplete treatment response in these dogs, the definitive cause remains unknown.

Reports of hookworm resistance are increasing in frequency in the USA and is a source of concern [29]. Since the initial report of hookworm resistance to pyrantel in racing greyhound populations, the problem has extended into the general dog population and has evolved to include resistance to macrocyclic lactones and benzimidazoles, in addition to pyrantel [29]. Combination anthelmintic therapy, targeting the same parasite with different modes of action, has long been used in farm animals to help fight resistance development [30]. Credelio Quattro combines two anthelmintics affecting larval, immature adult, and adult stages of *A. caninum* at different receptors, with moxidectin binding gamma-aminobutyric (GABA) and glutamate-gated chloride channels, and pyrantel binding ionophoric nicotinic acetylcholine receptors, causing worm death [29]. Credelio Quattro has demonstrated high efficacy in controlling hookworms across various geographies. In this US study, all remaining dogs in both groups confirmed highly robust efficacy with ≥ 97% reduction in fecal egg counts. However, veterinarians should remain vigilant by recommending regular environmental cleaning by pet owners and monitoring for *A. caninum* that is unresponsive to typical treatments through fecal diagnostic tests [29].

In dogs with a wide range of weight, age, gender, and breeds, Credelio Quattro was well tolerated and safe when administered alone and in combination with concomitant medications in this study. The most commonly reported adverse events for the Credelio Quattro-treated group was nonserious gastrointestinal signs, including diarrhea, emesis, and flatulence. These signs are consistent with adverse events reported in dogs with gastrointestinal infections and in previous studies evaluating Credelio Quattro. The animal rate of adverse events in this 10-d study was different than what was observed when these same products were utilized in a robust 11-month field study evaluating the prevention of heartworm disease. In this longer, more comprehensive study with a larger population of dogs, a more comparable occurrence of gastrointestinal adverse events was observed between the IVP and CP groups (diarrhea: 11% IVP, 8.3% CP; emesis: 9.4% IVP, 4.4% CP) [27].

## Conclusions

This multisite field study confirms the safety and effectiveness of a single dose of Credelio Quattro, a novel oral combination chewable tablet at the label dose ranges of 20–40 mg/kg lotilaner, 0.02–0.04 mg/kg moxidectin, 5–10 mg/kg praziquantel, and 5–10 mg/kg pyrantel, for the treatment and control of naturally occurring *A. caninum* and *T. canis* infections in dogs.

## Data Availability

Data supporting the conclusions of this article are included within the article.

## Abbreviations

CAPC: Companion Animal Parasite Council
CP: Control product
EHA: Egg hatch assay
EPG: Eggs per gram
FDA: Food and Drug Administration
GM: Geometric mean
L3: Infective third-stage larvae
IVP: Investigational veterinary product
LDA: Larval development assay
LS: Least squares
VLM: Visceral larva migrans
CLM: Cutaneous larval migrans

## References

[1] Drake J, Carey T. Seasonality and changing prevalence of common canine gastrointestinal nematodes in the USA. Parasit Vectors. 2019;12:430. 10.1186/s13071-019-3701-7. 31488192 10.1186/s13071-019-3701-7 PMC6728981

[2] Drake J, Parrish R. Dog importation and changes in canine intestinal nematode prevalence in Colorado, USA, 2013–2017. Parasit Vectors. 2020;13:404. 10.1186/s13071-020-04283-z. 32771048 10.1186/s13071-020-04283-z PMC7414742

[3] Holland CV. Knowledge gaps in the epidemiology of *Toxocara*: the enigma remains. Parasitology. 2017;144:81–94. 10.1017/S0031182015001407. 26670118 10.1017/S0031182015001407

[4] Strube C, Heuer L, Janecek E. *Toxocara* spp. infections in paratenic hosts. Vet Parasitol. 2013;193:375–89. 10.1016/j.vetpar.2012.12.033. 23312872 10.1016/j.vetpar.2012.12.033

[5] Campos-da-Silva DR, da Paz JS, Fortunato VR, Beltrame MA, Valli LC, Pereira FE. Natural infection of free-range chickens with the ascarid nematode *Toxocara* sp. Parasitol Res. 2015;114:4289–93. 10.1007/s00436-015-4669-7. 26319520 10.1007/s00436-015-4669-7

[6] Schnieder T, Laabs EM, Welz C. Larval development of *Toxocara canis* in dogs. Vet Parasitol. 2011;175:193–206. 10.1016/j.vetpar.2010.10.027. 21095061 10.1016/j.vetpar.2010.10.027

[7] Azam D, Ukpai OM, Said A, Abd-Allah GA, Morgan ER. Temperature and the development and survival of infective *Toxocara canis* larvae. Parasitol Res. 2012;110:649–56. 10.1007/s00436-011-2536-8. 21779864 10.1007/s00436-011-2536-8

[8] Al-Tae A-RA, Al-Bashir NM. The viability and infectivity of *Toxocara canis* infective larvae after a prolonged period of storage at different temperatures. MIRCEN J Appl Microbiol Biotechnol. 1988;4:349–55. 10.1007/BF01096140.

[9] Overgaauw PA. Aspects of *Toxocara* epidemiology: toxocarosis in dogs and cats. Crit Rev Microbiol. 1997;23:233–51. 10.3109/10408419709115138. 9347222 10.3109/10408419709115138

[10] Epe C. Intestinal nematodes: biology and control. Vet Clin North Am Small Anim Pract. 2009;39:1091–107. 10.1016/j.cvsm.2009.07.002. 19932365 10.1016/j.cvsm.2009.07.002

[11] Matsusake G, Takekawa T, Mogi K. Studies on the life history of the hookworms. X. The morphological studies on the development of the adult of *A. Caninum* in the normal host. II. Findings on 10–18 days old adult. Yokohama Med Bull. 1965;6:75–80. 14329147

[12] Hawdon JM, Wise KA. *Ancylostoma caninum* and other canine hookworms. In: Strube C, Mehlhorn H, editors. Dog parasites endangering human health. Parasitology research monographs, vol. 13. Cham: Springer; 2021.

[13] Bowman DD. Georgis’ parasitology for veterinarians. 11th ed. Amsterdam: Elsevier, Inc.; 2021.

[14] Stone WM, Girardeau M. Transmammary passage of *Ancylostoma caninum* larvae in dogs. J Parasitol. 1968;54:426–9. 5761146

[15] Schad GA, Page MR. *Ancylostoma caninum*: adult worm removal, corticosteroid treatment, and resumed development of arrested larvae in dogs. Exp Parasitol. 1982;54:303–9. 10.1016/0014-4894(82)90039-x. 7151941 10.1016/0014-4894(82)90039-x

[16] Kalkofen UP. Hookworms of dogs and cats. Vet Clin North Am Small Anim Pract. 1987;17:1341–54. 10.1016/s0195-5616(87)50005-5. 3328392 10.1016/s0195-5616(87)50005-5

[17] Bowman DD. Toxocara and toxocariasis, vol. 109. Cambridge: Academic Press; 2020.

[18] Hotez PJ, Brooker S, Bethony JM, Bottazzi ME, Loukas A, Xiao S. Hookworm infection. N Engl J Med. 2004;351:799–807. 10.1056/NEJMra032492. 15317893 10.1056/NEJMra032492

[19] Bowman DD, Montgomery SP, Zajac AM, Eberhard ML, Kazacos KR. Hookworms of dogs and cats as agents of cutaneous larva migrans. Trends Parasitol. 2010;26:162–7. 10.1016/j.pt.2010.01.005. 20189454 10.1016/j.pt.2010.01.005

[20] Companion Animal Parasite Council (CAPC): General Guidelines for Dogs and Cats. 2022. https://capcvet.org/guidelines/general-guidelines/. Accessed 02 Dec 2024.

[21] Mwacalimba K, Sears D, Brennan C, Poulsen Nautrup B, Sheehy J, Smith K, et al. Retrospective analyses of heartworm (*Diro**fi**laria immitis*) disease and ectoparasite preventive medication compliance in veterinary practices in the USA. Parasit Vectors. 2023;16:149. 10.1186/s13071-023-05735-y. 37106437 10.1186/s13071-023-05735-y PMC10142219

[22] Jimenez Castro PD, Venkatesan A, Redman E, Chen R, Malatesta A, Huff H, et al. Multiple drug resistance in hookworms infecting greyhound dogs in the USA. Int J Parasitol Drugs Drug Resist. 2021;17:107–17. 10.1016/j.ijpddr.2021.08.005. 34492564 10.1016/j.ijpddr.2021.08.005 PMC8426179

[23] Venkatesan A, Jimenez Castro PD, Morosetti A, Horvath H, Chen R, Redman E, et al. Molecular evidence of widespread benzimidazole drug resistance in *Ancylostoma caninum* from domestic dogs throughout the USA and discovery of a novel beta- tubulin benzimidazole resistance mutation. PLoS Pathog. 2023;19:e1011146. 10.1371/journal.ppat.1011146. 36862759 10.1371/journal.ppat.1011146 PMC10013918

[24] Leutenegger CM, Lozoya CE, Tereski J, Savard C, Ogeer J, Lallier R. Emergence of *Ancylostoma caninum* parasites with the benzimidazole resistance F167Y polymorphism in the US dog population. Int J Parasitol Drugs Drug Resist. 2023;21:131–40. 10.1016/j.ijpddr.2023.01.001. 36958067 10.1016/j.ijpddr.2023.01.001 PMC10068012

[25] Jimenez Castro PD, Howell SB, Schaefer JJ, Avramenko RW, Gilleard JS, Kaplan RM. Multiple drug resistance in the canine hookworm *Ancylostoma caninum*: an emerging threat? Parasit Vectors. 2019;12:576. 10.1186/s13071-019-3828-6. 31818311 10.1186/s13071-019-3828-6 PMC6902405

[26] Vercruysse J, Holdsworth P, Letonja T, Conder G, Hamamoto K, Okano K, et al. International harmonisation of anthelmintic efficacy guidelines (part 2). Vet Parasitol. 2002;103:277–97. 10.1016/s0304-4017(01)00615-x. 11777607 10.1016/s0304-4017(01)00615-x

[27] FDA CVM: Credelio Quattro NADA 141–581. 2024.

[28] Charles S, Deuster K, Wang X, et al. Efficacy of a novel chewable tablet (Credelio Quattro™) containing lotilaner, moxidectin, praziquantel, and pyrantel for the treatment and control of hookworm infections in dogs. Parasites Vectors. 2025;18:125. 10.1186/s13071-025-06757-4. 40176170 10.1186/s13071-025-06757-4 PMC11963408

[29] Marsh AE, Lakritz J. Reflecting on the past and fast forwarding to present day anthelmintic resistant *Ancylostoma caninum*—a critical issue we neglected to forecast. Int J Parasitol Drugs Drug Resist. 2023;1:36–43. 10.1016/j.ijpddr.2023.04.003. 10.1016/j.ijpddr.2023.04.003 PMC10229760 37229949

[30] Bartram DJ, Leathwick DM, Taylor MA, Geurden T, Maeder SJ. The role of combination anthelmintic formulations in the sustainable control of sheep nematodes. Vet Parasitol. 2012;186:151–8. 10.1016/j.vetpar.2011.11.030. 22245073 10.1016/j.vetpar.2011.11.030

